# Supplementary material for: A deep learning model to predict RNA-Seq expression of tumours from whole slide images
Source: Nat Commun. 2020 Aug 3;11:3877. doi: 10.1038/s41467-020-17678-4 (PMC7400514; doi:10.1038/s41467-020-17678-4)
Supplement: Supplementary file 1 — Supplementary Information [file 41467_2020_17678_MOESM1_ESM.pdf]

A deep learning model to predict RNA-seq expression of tumours from whole slide images

Benoît Schmauch[1], Alberto Romagnoni[1,^], Elodie Pronier[1,^], Charlie Saillard[1], Pascale Maillé[2], Julien Calderaro[2], Aurélie Kamoun[1], Meriem Sefta[1], Sylvain Toldo[1], Mikhail Zaslavskiy[1], Thomas Clozel[1], Matahi Moarii[1], Pierre Courtiol[1,#], Gilles Wainrib[1,#]

[1]: Owkin Lab, Owkin, Inc. New York, NY USA.

[2]: INSERM U955, Team "Pathophysiology and Therapy of Chronic Viral Hepatitis and Related Cancers", Créteil, France; APHP, Department of Pathology, Hôpital Henri Mondor, Université Paris-Est, Créteil, France.

[^,#]: These authors contributed equally.

Corresponding authors:

Gilles Wainrib:

Owkin Lab, Owkin, Inc. New York, NY, 10003 USA.

gilles.wainrib@owkin.com

Benoît Schmauch:

Owkin Lab, Owkin, Inc. New York, NY, 10003 USA.

benoit.schmauch@owkin.com

Supplementary Information

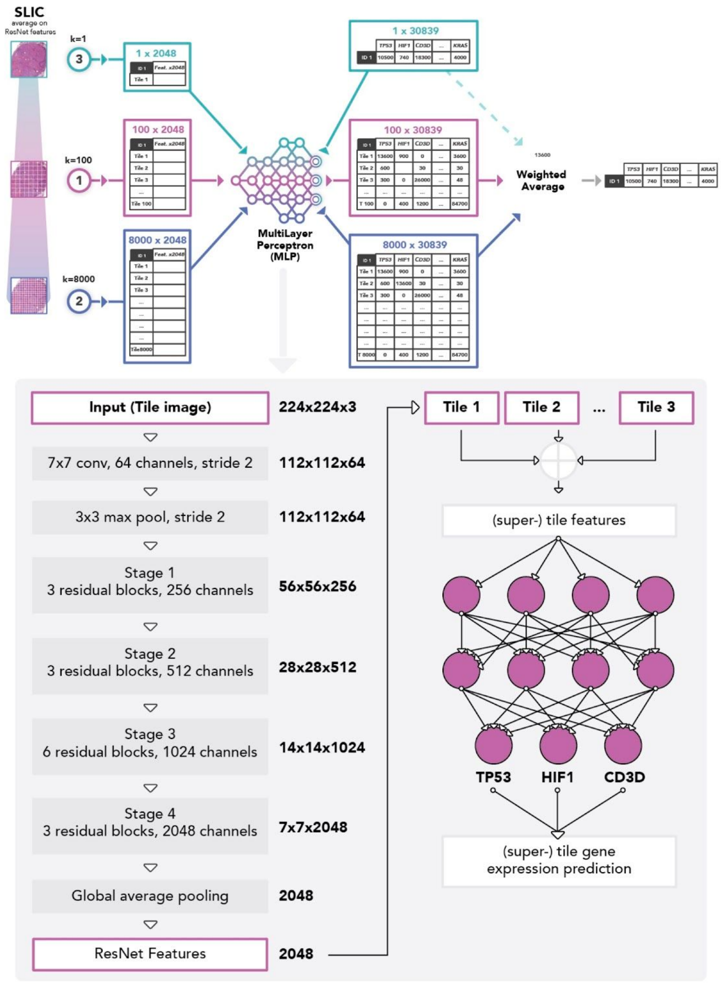

Supplementary Figure 1. Preprocessing and model structure. The whole-slide image is divided into 112 × 112 µm tiles (224 × 224 pixels). A maximum of 8,000 such tiles (excluding those covering the background) are kept. A 50-layer ResNet, pretrained on the ImageNet dataset, is used to extract 2,048 features from each tile image (bottom left). A preprocessing algorithm, inspired by simple linear iterative clustering (SLIC) is applied to the 8,000 tiles to produce *k* super-tiles. An average is obtained for each super-tile, at the level of the 2,048 ResNet features. The number of clusters is decided according to the task (color and numerical code as in Fig. 1). A multilayer perceptron (bottom right) is applied to each cluster of the slide. The last layer of the model encodes the transcriptomic representation described in the text. This representation is then used to produce a prediction per cluster and per Ensembl gene of the RNA-Seq dataset. Finally, a weighted average (described in the Methods) provides the output prediction of gene expression associated with the slide.

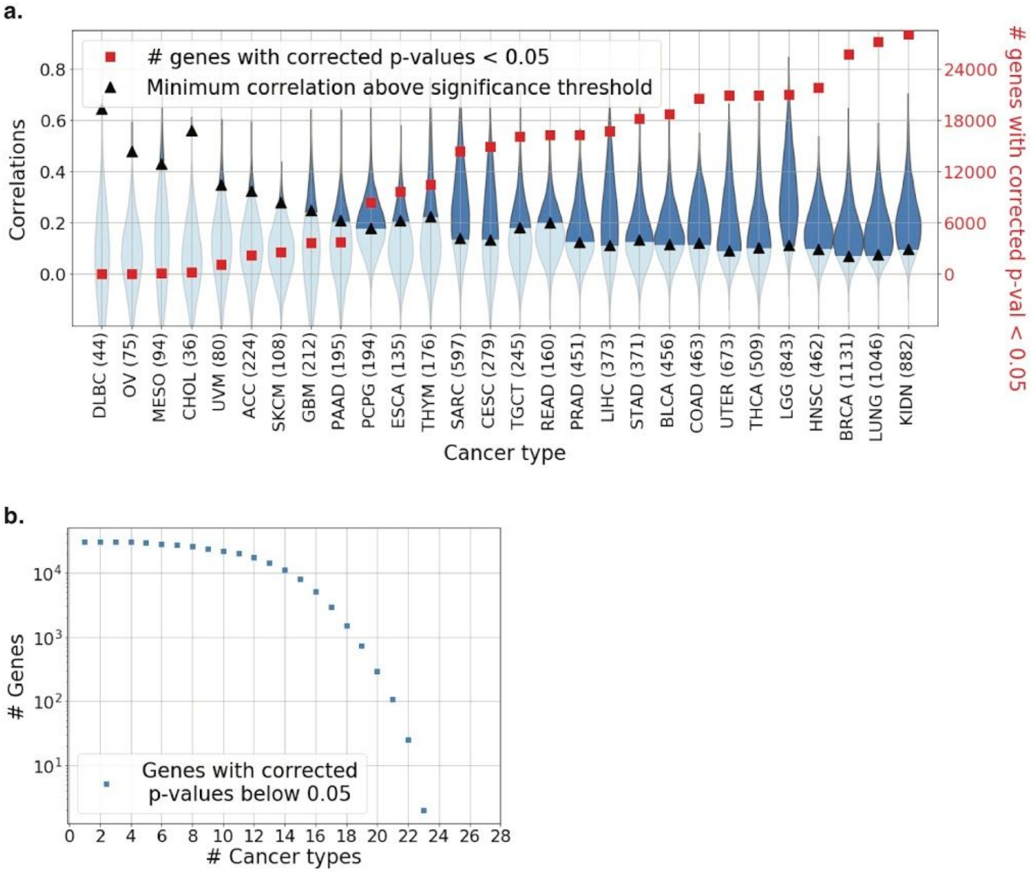

Supplementary Figure 2. Predictions of gene expression after Benjamini–Hochberg correction for the testing of multiple hypotheses. a. Distribution of Pearson's correlation coefficients *R* (left axis, blue violin plots) and the number of coding and non-coding genes (right axis, red squares) using Benjamini–Hochberg-corrected p-values < 0.05 (one-sided empirical p-value, as described in Methods section), for twenty eight cancer types from the TCGA. Black triangles indicate the minimal correlation coefficient required for significance in any given dataset. **b.** Number of coding and non-coding genes for which expression was significantly well-predicted for a given number of cancer types, as a function of the number of cancers.

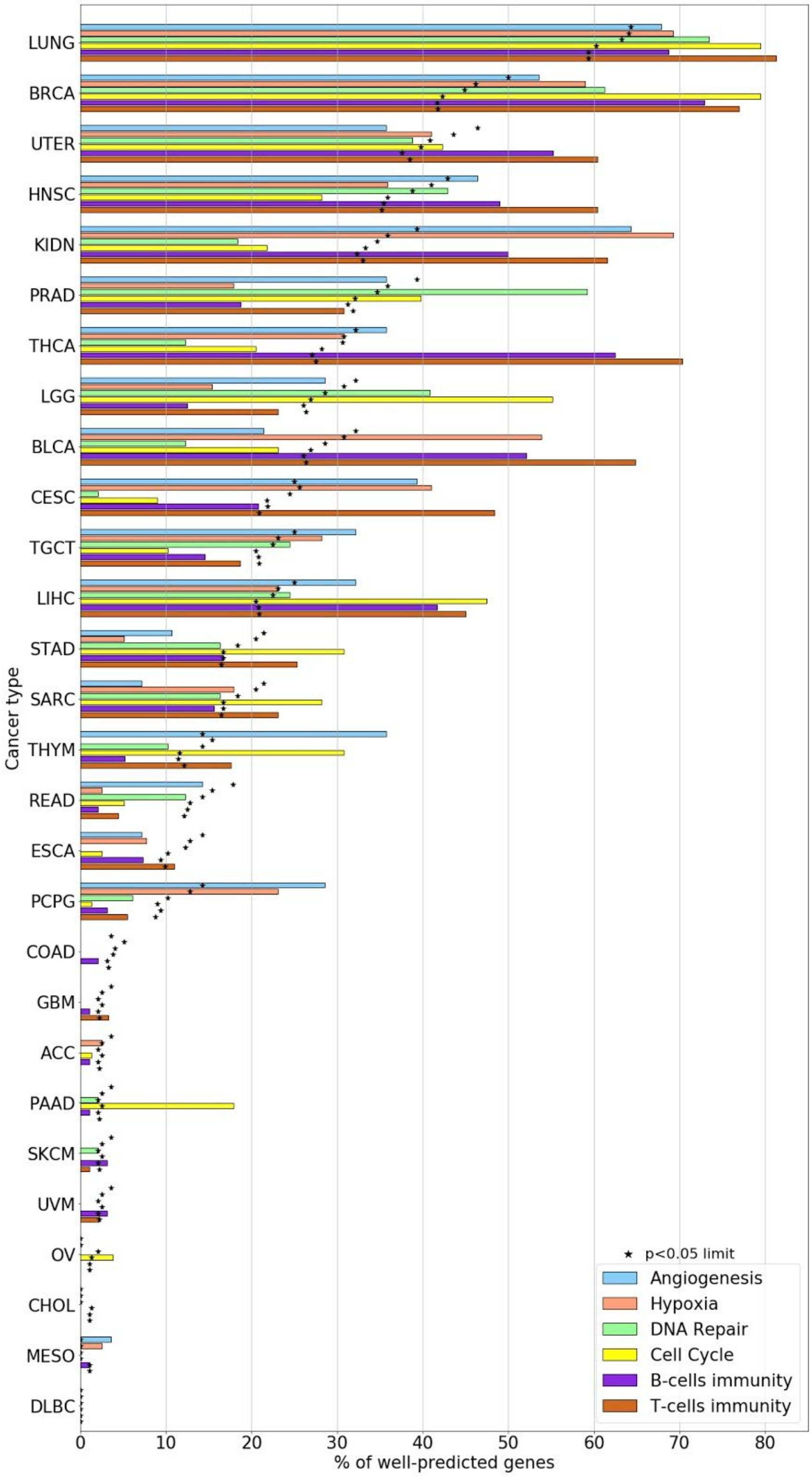

Supplementary Figure 3. Percentage of genes for which expression was well-predicted in six common pathways of carcinogenesis. Percentage of genes for which expression was well-predicted (as in Fig.2) for the six studied hallmark pathways for cancer. The black stars indicate the percentage required for each cancer dataset and each pathway to be considered significantly better predicted than a corresponding random list of genes of the same length as the pathway gene list (see Methods section).

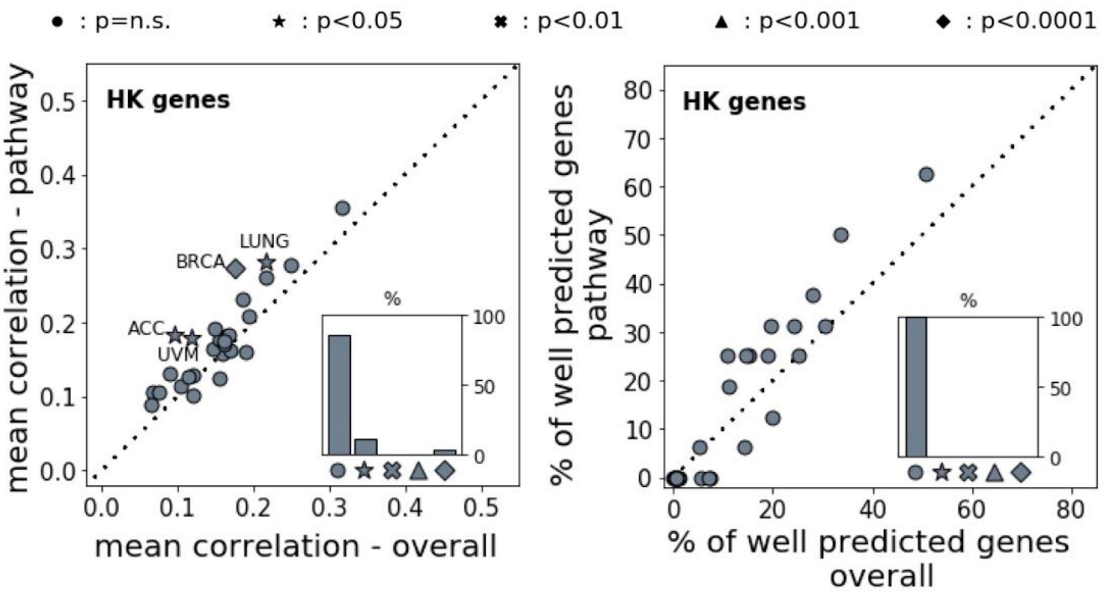

Supplementary Figure 4. Prediction of a housekeeping genes signature. As in Fig.3, for the housekeeping gene signature described in Supplementary Table 4. *Left panel.* The indicated statistical significance refers to the probability of obtaining a correlation $R > R_p$ in the distribution of correlations for random lists, for each given cancer type. Insets show the percentages of the different cases of statistical significance between cancer types. *Right panel*. As in the left panel, but for the percentage of genes for which expression was of well-predicted (as defined in the text and in Fig. 2). HK = housekeeping.

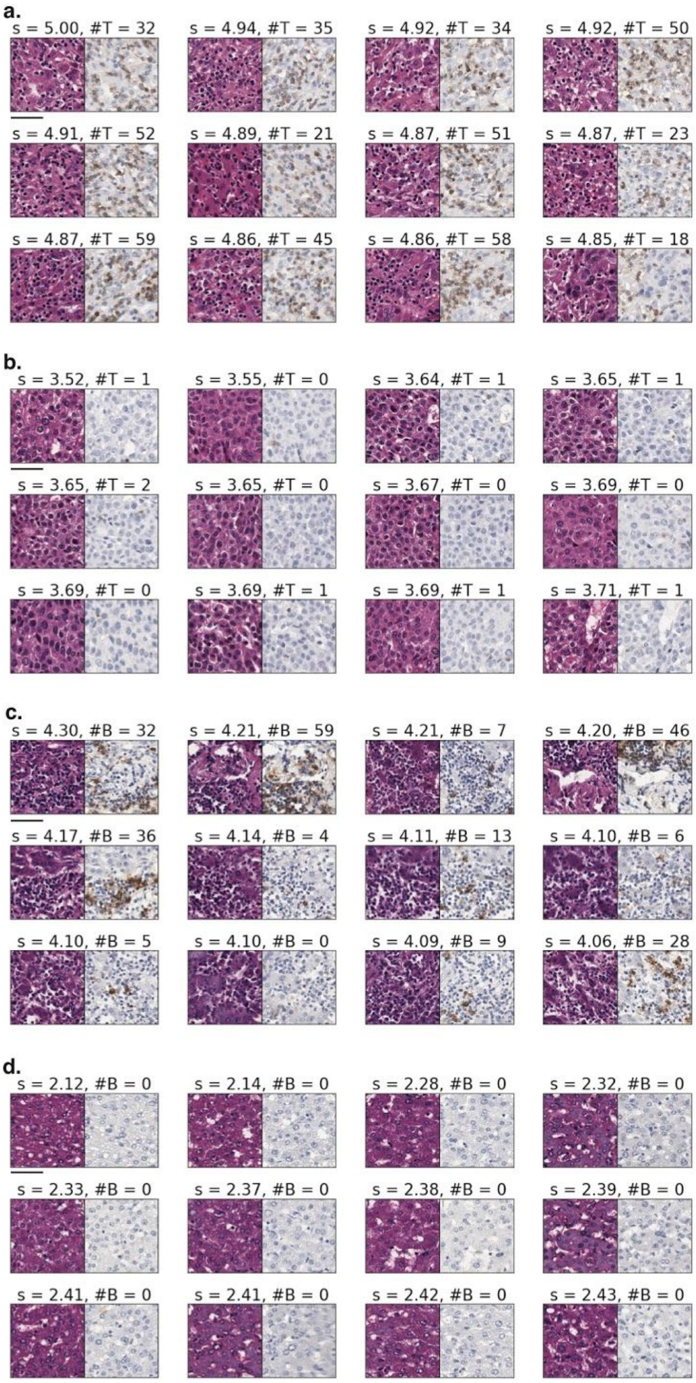

Supplementary Figure 5. Most predictive tiles for CD3-receptor encoding genes. a. Extraction of the tiles associated with the highest score for $CD3^{+}$-encoding genes expression predicted from the HE/CD3 slide. S = Score corresponding to the log expression score of each tile; #T= Number of T cells per tile as determined with QuPath from the CD3 IHC. **b.** As in **a** but for the tiles with the lowest score for CD3 expression. **c.** Extraction of the tiles associated with the highest score for *CD19* and *CD20* expression predicted from the HE/CD20 slide. S = Score corresponding to the log expression score of each tile; #B= Number of B cells per tile as determined with QuPath from the CD20 IHC. **d.** As in **b** but for the tiles with the lowest score for CD3 expression. Scale bar: 100µm. Representative tiles from one double-stained slide (n=28,123 tiles)

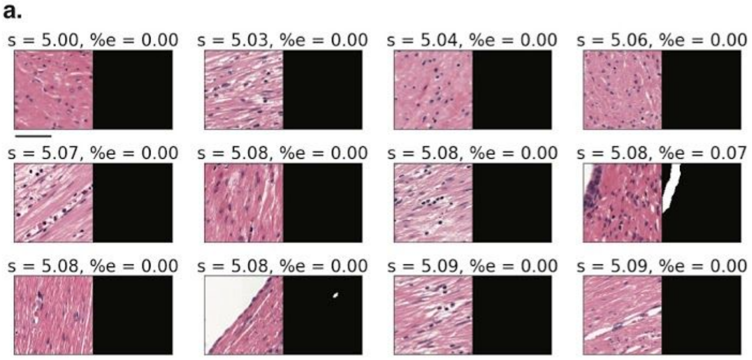

Supplementary Figure 6. Least predictive tiles for epithelium associated genes. Extraction of the tiles associated with the lowest score for epithelium-associated genes expression from the same slide as in Fig. 5. S = Score corresponding to the log expression score of each tile; %e = fraction of pixels marked as belonging to the epithelium. Scale bar: 100µm. (*n*= 21,714 tiles).

a.

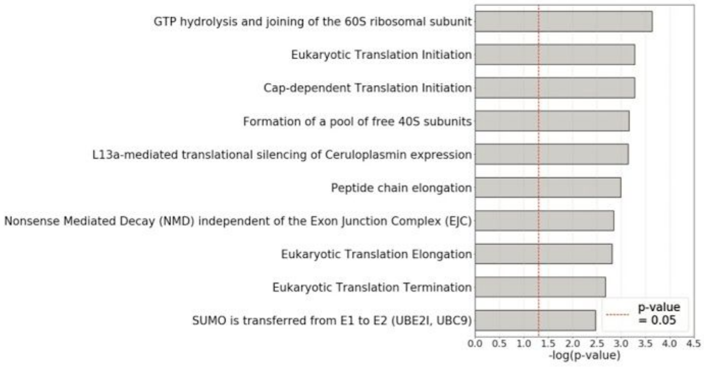

Supplementary Figure 7: Computational pathway analysis of the best-predicted genes among the microsatellite stable patients of the TCGA-CRC cohort.

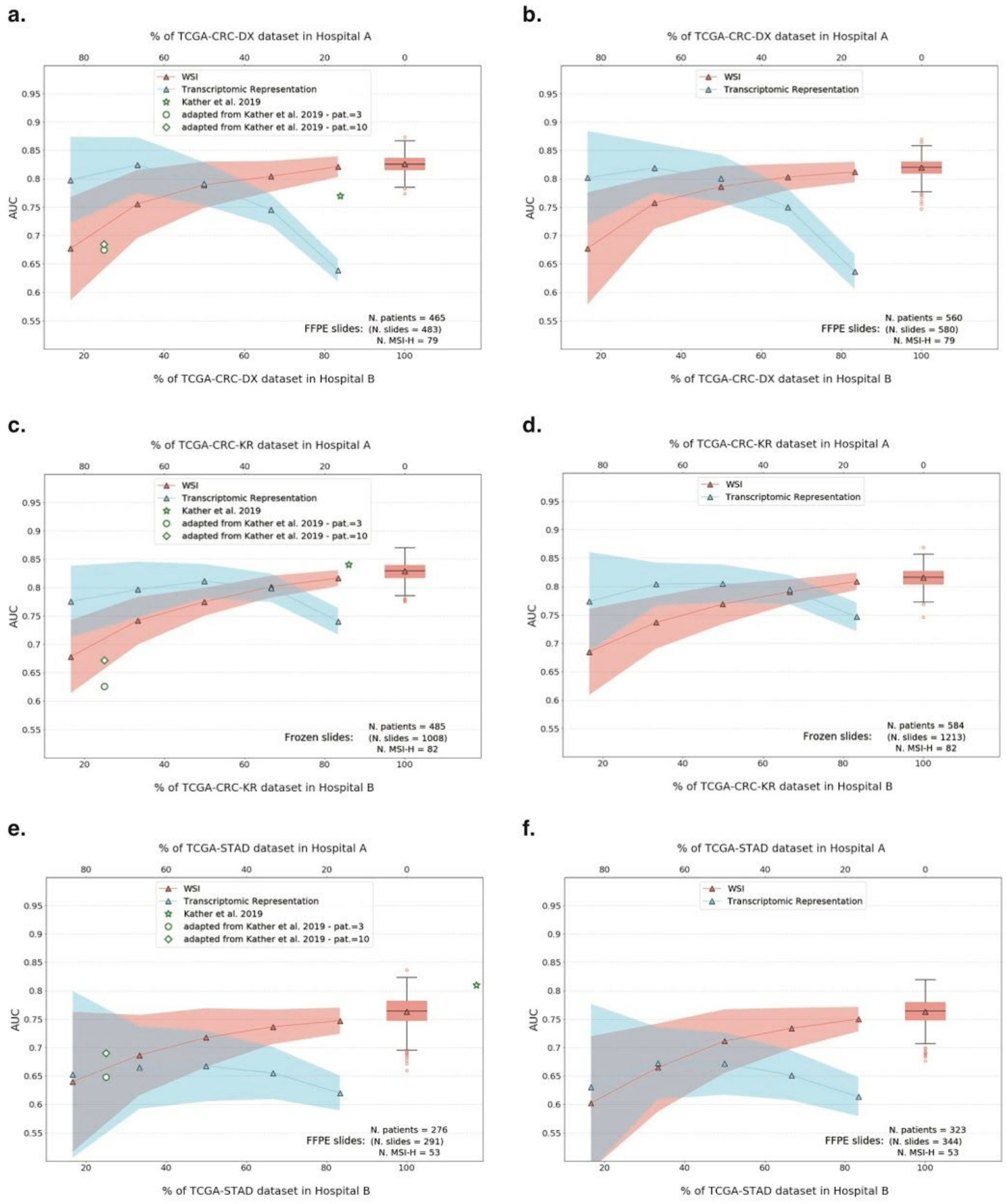

Supplementary Figure 8. Prediction of microsatellite instability status using transfer learning from transcriptomic representation. Change in area under the ROC curve (AUC) for the model based on the transcriptomic representation learned in Hospital A and trained on Hospital B (blue) and the model directly based on WSI images from Hospital B (red), as a function of the fraction of the dataset used in the two hospitals, for the different datasets used in the present study, with or without MSI-Low (MSI-L) patients included in the negative class together with MSS patients (*n* = 50 different data splits between the hospitals, averaged over 10 different 3-folds CV; solid lines and triangles: mean over splits; shaded area: 68% confidence interval). Whenever comparison is possible, we also display the result reported by Kather et al.[55] and that obtained from adapting the same method with 25% of the data in Hospital B (see Methods section for further details). **a.** and **b.** TCGA-CRC-DX, respectively without and with MSI-L patients.

c. and **d.** TCGA-CRC-KR, resp. without and with MSI-L patients. **e.** and **f.** TCGA-STAD, resp. without and with MSI-L patients. Boxplot in each panel represents the distribution of AUC values (500 3-folds CV) over the whole dataset, for the model based on WSI images (box: interquartile range (IQR); horizontal line: median; whiskers: 1.5 times IQR, triangle: mean; open circles: outliers).

Project ID	Disease Name	# Patients	# Samples
BRCA	Invasive breast carcinoma	1057	1131
LUNG	Lung adenocarcinoma (LUAD)	944	1046
	Lung squamous cell carcinoma (LUSC)		
KIDN	Kidney chromophobe carcinoma (KICH)	843	882
	Kidney renal clear cell carcinoma (KIRC)		
	Kidney renal papillary cell carcinoma (KIRP)		
LGG	Brain lower-grade glioma	485	843
SARC	Sarcoma	252	597
UTER	Uterine carcinosarcoma (UCS)	558	673
	Uterine corpus endometrial carcinoma (UCEC)		
THCA	Thyroid carcinoma	497	509
COAD	Colon adenocarcinoma	445	463
HNSC	Head and neck squamous cell carcinoma	423	462
BLCA	Bladder urothelial carcinoma	383	456
PRAD	Prostate adenocarcinoma	399	451
LIHC	Liver hepatocellular carcinoma	359	373
STAD	Stomach adenocarcinoma	350	371
CESC	Cervical squamous cell carcinoma and endocervical adenocarcinoma	267	279
TGCT	Testicular germ cell tumors	149	245
ACC	Adrenocortical carcinoma	55	224

GBM	Glioblastoma multiforme	96	212
PAAD	Pancreatic adenocarcinoma	175	195
PCPG	Pheochromocytoma and paraganglioma	175	194
THYM	Thymoma	116	176
READ	Rectum adenocarcinoma	159	160
ESCA	Esophageal carcinoma	133	135
SKCM	Skin, cutaneous melanoma	97	108
MESO	Mesothelioma	74	94
UVM	Uveal melanoma	80	80
OV	Ovarian serous cystadenocarcinoma	74	75
DLBC	Lymphoid neoplasm diffuse large B cell lymphoma	44	44
CHOL	Cholangiocarcinoma	36	36

Supplementary Table 1: TCGA dataset, detailed information. A matched WSI RNA-Seq data pair is considered here to be a sample.

Rank of tile prediction	n_{tiles} = 100	n_{tiles} = 8,000
1	0.269	0.0210
1 to 2	0.126	
2 to 5	0.054	
5 to 10	0.026	
10 to 20	0.011	0.0099
20 to 50	0.004	0.0043
50 to 100	0.001	0.0021
100 to 200	-	0.0010
200 to 500	-	0.0004
500 to 1,000	-	0.0002
1,000 to 2,000	-	8×10^{-5}
2,000 to 5,000	-	2×10^{-5}

Supplementary Table 2: Coefficients of tile predictions in the weighted sum. These coefficients are defining the slide-level gene expression predicted by the model during inference, as a function of their rank.

Angiogenesis	HALLMARK_ANGIOGENESIS,<br>BIOCARTA_VEGF_PATHWAY,<br>KEGG_VEGF_SIGNALING_PATHWAY,<br>GO_ANGIOGENESIS
Hypoxia	HALLMARK_HYPOXIA,<br>BIOCARTA_HIF_PATHWAY,<br>GO_REGULATION_OF_CELLULAR_RESP<br>ONSE_<br>TO_HYPOXIA
DNA repair	HALLMARK_DNA_REPAIR,<br>REACTOME_DNA_REPAIR,<br>GO_DNA_REPAIR
Cell cycle	BIOCARTA_CELLCYCLE_PATHWAY,<br>KEGG_CELL_CYCLE,<br>REACTOME_CELL_CYCLE,<br>GO_CELL_CYCLE
B cell-mediated immunity	GO_B_CELL_MEDIATED_IMMUNITY
T cell-mediated immunity	REACTOME_ADAPTATIVE_IMMUNE_<br>SYSTEM<br>GO_ADAPTATIVE_IMMUNE_RESPONSE<br>GO_REGULATION_OF_ADAPTATIVE_IMM<br>UNE_RESPONSE

Supplementary Table 3: List of signatures from GSEA combined to define the six cancer pathways.

Ingenuity Canonical Pathways	-log(p-value)	Ratio	Genes
Th1 and Th2 Activation Pathway	14.1	0.0909	*CD247, CCR1, IL2RG, CD3E, IL12RB1, HAVCR2, CXCR3, CD8A, CD3D, CD3G, PIK3CG, IL10RA, CD86, IL2RA, VAV1, HLA-DPB1, HLA-DPA1*
iCOS-iCOSL Signaling in T Helper Cells	12.9	0.112	*PTPRC, CD247, CD3G, IL2RG, LCK, CD3E, PIK3CG, ZAP70,TRAT1, CD86, IL2RA, VAV1, CD3D, ITK*
Th1 Pathway	12.4	0.102	*CD247, CD3E, IL12RB1, HAVCR2, CXCR3, CD8A, CD3D, CD3G, PIK3CG, IL10RA, CD86, VAV1, HLA-DPB1, HLA-DPA1*
T Cell Receptor Signaling	10.6	0.1	*PTPRC, CD247, CD3G, LCK, PTPN7, CD3E, PIK3CG, ZAP70, VAV1, CD8A, CD3D, ITK*
Th2 Pathway	10.5	0.0855	*CCR1, CD247, CD3G, IL2RG, CD3E, IL12RB1, PIK3CG, CD86, IL2RA, VAV1, HLA-DPB1, CD3D, HLA-DPA1*
CD28 Signaling in T Helper Cells	10	0.0896	*PTPRC, CD247, CD3G, LCK, CD3E, WAS, PIK3CG, ZAP70, CD86, VAV1, CD3D, ITK*
Primary Immunodeficiency Signaling	8.87	0.16	*PTPRC, IL2RG, LCK, CD3E, ZAP70, CIITA, CD8A, CD3D*
CTLA4 Signaling in Cytotoxic T Lymphocytes	8.84	0.099	*CD247, CD3G, LCK, CD3E, PIK3CG, ZAP70, TRAT1,CD86 CD8A, CD3D*
Pathogenesis of Multiple Sclerosis	8.82	0.556	*CCR1, CCL4, CXCR3, CCL5, CXCL9*
Natural Killer Cell Signaling	7.68	0.0752	*CD247, LCK, SH2D1A, LAIR1, TYROBP, PIK3CG, ZAP70, VAV1, HCST, FCGR3A/FCGR3B*

Supplementary Table 4. Pathways well-predicted in at least 12 cancer types. Canonical pathways with the best overlap with the 156 genes for which expression was well-predicted in at least 12 cancer types. P-values were calculated using Right-Tailed Fisher's Exact Test.

Ingenuity Canonical Pathways	-log(p-value)	Ratio	Genes
Cell Cycle Control of Chromosomal Replication	10.6	0.286	*MCM6, CDC45, CDT1, CDK16, CDC6, ORC6, CDC7, CDK1, MCM4, MCM3, MCM2, TOP2A, PRIM2, DBF4, ORC1, MCM7*
Mitotic Roles of Polo-Like Kinase	8.41	0.227	*KIF23, CDC20, PTTG1,PRC1, CDC7, CCNB2, PLK1, CDK1, CCNB1, PLK4, TGFB1, FBXO5, PKMYT1, KIF11, CDC25A*
Hepatic Fibrosis / Hepatic Stellate Cell Activation	8.39	0.134	*COL8A2, CCR5, COL10A1, COL4A2, COL1A2, COL5A1, COL16A1, TIMP1, TGFB1, PDGFRA, TIMP2, CXCL8, COL6A2, FGFR2, MMP2, COL1A1, COL6A3, TGFB3, TGFA, IL10RA, EDNRA, COL11A1, COL9A2, MMP9, COL3A1*
Th1 and Th2 Activation Pathway	7.68	0.128	*CCR5, IL2RG, HLA-DOA, CD3E, IL12RB1, IKZF1, HAVCR2, PIK3R5, HLA-DQA1, LGALS9, FGFR2, SPI1, CD3G, IL18, TGFB1, HLA-DMB, IL10RA, CD86, IL2RA, VAV1, JAG1, JAK3 ,NOTCH1, HLA-DPA1*
GP6 Signaling Pathway	6.85	0.141	*COL8A2,COL6A2,PIK3R5,COL10A1,FGFR2,COL4A2,LAMC2,COL16A1,COL5A1,COL1A2,COL1A1,COL6A3,SYK,LAMB1,FCER1G,COL11A1,COL9A2,LCP2,COL3A1*
Th2 Pathway	6.69	0.132	*CCR5, IL2RG, HLA-DOA, CD3E, IL12RB1, IKZF1, PIK3R5, HLA-DQA1, FGFR2, SPI1, CD3G, TGFB1, HLA-DMB, CD86, IL2RA, VAV1, JAG1, JAK3, NOTCH1, HLA-DPA1*
Th1 Pathway	6.07	0.131	*CCR5, HLA-DOA,CD3E, IL12RB1, HAVCR2, PIK3R5, HLA-DQA1, LGALS9, FGFR2, CD3G, IL18, HLA-DMB, IL10RA, CD86, VAV1, JAK3, NOTCH1, HLA-DPA1*
Role of BRCA1 in DNA Damage Response	5.59	0.162	*RAD51, FANCB, FANCD2, RFC4, FANCG, BARD1, SMARCD1, PLK1, E2F3, BLM, RBL1, E2F2, CHEK1*
CD28 Signaling in T Helper Cells	5.56	0.127	*HLA-DOA, ARPC1B, CD3E, PIK3R5, HLA-DQA1, FGFR2, IKBKE, CD3G, LCK, CARD11, SYK, ITPR3, HLA-DMB, FCER1G, CD86, VAV1, LCP2*
iCOS-iCOSL Signaling in T Helper Cells	5.32	0.128	*HLA-DOA, IL2RG, CD3E, HLA-DQA1, PIK3R5, FGFR2, IKBKE, CD3G, LCK, HLA-DMB, ITPR3, FCER1G, CD86, VAV , IL2RA, LCP2*

Supplementary Table 5. Pathways well-predicted in hepatocellular carcinoma samples. Canonical pathways with the best overlap with the genes for which expression was best predicted (correlation coefficient above 0.4) in liver hepatocellular carcinomas. P-values were calculated using Right-Tailed Fisher's Exact Test.

Ingenuity Canonical Pathways	-log(p-value)	Ratio	Genes
Primary Immunodeficiency Signaling	16.3	0.4	*CD19, IL2RG, CD3E, IGLL1/IGLL5, CIITA,CD79A, IGHG1, CD8A,TNFRSF13C, CD3D, TAP1, IL7R, LCK, IGHG3, ICOS, ZAP70, IGHM, IGHA1, JAK3, TAP2*
Cell Cycle Control of Chromosomal Replication	12.8	0.321	*MCM5, MCM6, CDC45, CDT1, CDC6, CDC7, ORC6, CDK1, MCM4, MCM3, PCNA, MCM2, TOP2A, PRIM2, CHEK2, DBF4, MCM7, ORC1*
Mitotic Roles of Polo-Like Kinase	10.4	0.258	*KIF23,CDC25C, ESPL1, CDC20, PTTG1, PRC1, CDC7, CCNB2, PLK1, CDK1, CCNB1, CDC25B, PLK4, FBXO5, CHEK2, KIF11, CDC25A*
Th1 and Th2 Activation Pathway	9.73	0.144	*CD247,CD3E,KLRD1,IL12RB1,CXCR3,CD8A, TBX21,IL18R1,IL2RB,RUNX3,IFNG,IL2RG,IKZF1,IL12RB2,CD3D,STAT4,CD3G,LTA,ICOS, GFI1,CXCR6,S1PR1,APH1B,HLA-DOB,IL2RA,PIK3CD,JAK3*
Cell Cycle: G2/M DNA Damage Checkpoint Regulation	9.32	0.286	*CDC25C, CKS2, YWHAZ, CCNB2, PLK1, AURKA, CDK1, CHEK1, SKP2, CCNB1, CDC25B, CKS1B, TOP2A, CHEK2*
Role of CHK Proteins in Cell Cycle Checkpoint Control	8.37	0.246	*CDC25C, PLK1, E2F3, CDK1, CHEK1, PCNA, RFC4, E2F1, RFC2, CLSPN, E2F2, CHEK2, E2F8, CDC25A*
Estrogen-mediated S-phase Entry	8.24	0.385	*CCNA2, CCNE1, E2F1, E2F3, ESR1, E2F8, E2F2, CDK1, SKP2, CDC25A*
Th2 Pathway	8.07	0.145	*CD247, RUNX3, IFNG, IL2RG, CD3E, IL12RB1, IKZF1, IL12RB2, TBX21, CD3D, STAT4, CD3G, ICOS, GFI1, CXCR6, S1PR1, APH1B, HLA-DOB, IL2RA, PIK3CD, JAK3, IL2RB*
Th1 Pathway	7.46	0.146	*CD247, RUNX3, IFNG, CD3E, IL12RB1, KLRD1, CXCR3, IL12RB2, TBX21, CD8A, CD3D, IL18R1, STAT4, CD3G, LTA, ICOS, APH1B, HLA-DOB, PIK3CD, JAK3*
T Helper Cell Differentiation	6.9	0.192	*IL6ST, IFNG, IL2RG, IL12RB1, IL21R, FOXP3, IL12RB2, TBX21, IL18R1, STAT4, ICOS, HLA-DOB, IL2RA,TNFRSF1B*

Supplementary Table 6. Pathways well-predicted in breast cancer samples. Canonical pathways with the best overlap with the genes for which expression was best predicted (correlation coefficient above 0.4) in breast cancer samples. P-values were calculated using Right-Tailed Fisher's Exact Test.

Angio genesis	Hypoxia	DNA repair	Cell cycle	B cells	T cells	Housekeep ing
CDC42	*ADM*	*CETN2*	*ATM*	*MASP2*	*CD79B*	*RPL32*
FGFR1	*ALDOA*	*DDB1*	*ATR*	*IGLC7*	*BTLA*	*PPIA*
FLT1	*BHLHE40*	*DDB2*	*BUB1*	*IGLC3*	*WAS*	*PGK1*
FLT4	*BNIP3L*	*ERCC1*	*BUB1B*	*IGKV3-20*	*CTSC*	*HMBS*
HIF1A	*CA12*	*ERCC2*	*BUB3*	*C3*	*ZAP70*	*GAPDH*
HRAS	*CCNG2*	*ERCC3*	*CCNA1*	*IGHG4*	*FYN*	*GUSB*
ITGAV	*CDKN1A*	*ERCC4*	*CCNA2*	*IGKV4-1*	*ANXA1*	*TBP*
JAG1	*CDKN1B*	*ERCC5*	*CCNB1*	*CD74*	*IFNG*	*PSMB2*
KDR	*COL5A1*	*ERCC8*	*CCNB2*	*C8A*	*PVR*	*ALB*
MAPK14	*CP*	*FEN1*	*CCND1*	*IGLL1*	*C3*	*HPRT1*
NRP1	*DDIT3*	*GTF2H1*	*CCND2*	*MLH1*	*LILRB1*	*EMC7*
NOS3	*EDN2*	*GTF2H3*	*CCND3*	*SERPING1*	*HRAS*	*RPS27*
NFATC4	*ENO1*	*GTF2H5*	*CCNE1*	*POU2F2*	*CD74*	*RPLP0*
PIK3CA	*F3*	*LIG1*	*CCNE2*	*LIG4*	*TRAF6*	*SDHA*
PIK3CB	*FOS*	*MPG*	*CCNH*	*HLA-DRB1*	*LILRB5*	*ACTB*
PIK3CG	*GAPDH*	*PCNA*	*CDC14A*	*CFI*	*HLA-B*	*AC010970.1*
PIK3R1	*HIF1A*	*POLB*	*CDC16*	*IL4R*	*FCGR1B*	
PDGFA	*IGFBP3*	*POLD1*	*CDC20*	*CD40LG*	*PRKCQ*	
PRKCA	*HK1*	*POLD3*	*CDC23*	*BCL3*	*HLA-DRB1*	
PRKCB	*HK2*	*POLD4*	*CDC25A*	*IGLC6*	*ERAP1*	
PLCG1	*HMOX1*	*POLH*	*CDC25B*	*RNF8*	*IL4R*	
PTK2	*IGFBP1*	*POLL*	*CDC25C*	*IGHM*	*RIPK2*	
PTGS2	*IL6*	*POLR2A*	*CDC26*	*HLA-DQB1*	*IL4*	
PXN	*JUN*	*POLR2C*	*CDC27*	*C4BPB*	*CD40LG*	
SHC1	*LDHA*	*POLR2D*	*CDC45*	*BATF*	*HMGB1*	

SH2D2A	*MIF*	*POLR2E*	*CDC6*	*ZP3*	*KLRK1*	
VEGFA	*P4HA1*	*POLR2F*	*CDC7*	*IGHG3*	*CTLA4*	
VAV2	*PDGFB*	*POLR2G*	*CDK1*	*SWAP70*	*LILRB2*	
	PFKL	*POLR2H*	*CDK2*	*C1QBP*	*PTPRC*	
	PFKP	*POLR2I*	*CDK4*	*BCL10*	*HAVCR2*	
	PGF	*POLR2J*	*CDK6*	*FAS*	*C4BPB*	
	PGK1	*POLR2K*	*CDK7*	*EXO1*	*SLAMF1*	
	PLAUR	*RAD51*	*CDKN1A*	*IGLC2*	*ZP3*	
	SLC2A1	*RAD52*	*CDKN1B*	*IGHG2*	*LILRA1*	
	SLC2A3	*RBX1*	*CDKN2A*	*IGKV2-40*	*TRAT1*	
	STC1	*REV3L*	*CDKN2B*	*CD55*	*STAT6*	
	TGFB3	*RFC2*	*CDKN2C*	*PRKCD*	*PTPN6*	
	TGM2	*RFC3*	*CDKN2D*	*INPP5D*	*CTSS*	
	VEGFA	*RFC4*	*CHEK1*	*AICDA*	*BCL10*	
		RFC5	*CHEK2*	*IGHV4OR1 5-8*	*LYN*	
		RPA2	*CUL1*	*IL13RA2*	*TGFB1*	
		RPA3	*DBF4*	*C4A*	*TAP2*	
		TP53	*E2F1*	*C1R*	*TRPM4*	
		XPC	*E2F2*	*IGHA2*	*CD8A*	
		ATM	*E2F3*	*C4BPA*	*SYK*	
		ATR	*E2F4*	*NBN*	*CTSH*	
		CHEK1	*E2F5*	*IGHG1*	*PIK3CD*	
		CHEK2	*HDAC1*	*C7*	*FOXP3*	
		H2AFX	*KI67*	*MBL2*	*CTSL*	
			MAD1L1	*IGHV2-70*	*PAG1*	
			MAD2L1	*C1QA*	*NECTIN2*	
			MCM2	*HSPD1*	*CD79A*	

			MCM3	*C1RL*	*C4BPA*	
			MCM4	*IGKV1D-33*	*TNFSF18*	
			MCM5	*C9*	*CRTAM*	
			MCM6	*MSH6*	*BTK*	
			MCM7	*C8G*	*IL2*	
			MDM2	*IGLL5*	*RAET1E*	
			MYC	*IGLV7-43*	*HSPD1*	
			ORC1	*IRF7*	*HLA-A*	
			PCNA	*CD27*	*MSH6*	
			PKMYT1	*GCNT3*	*TNFRSF13C*	
			PLK1	*C6*	*EIF2AK4*	
			PTTG1	*ERCC1*	*HLA-E*	
			RAD21	*IGKV3D-20*	*IRF7*	
			RB1	*EXOSC3*	*PRKCB*	
			RBL1	*FCER1G*	*CD4*	
			RBL2	*C1S*	*EXOSC3*	
			SKP1	*IGKV1-5*	*IL12B*	
			SKP2	*MSH2*	*FCER1G*	
			SMC1A	*IGKC*	*JAK3*	
			SMC1B	*GAPT*	*B2M*	
			SMC3	*C1QC*	*BCL6*	
			STAG1	*CR2*	*LILRB3*	
			STAG2	*RNF168*	*MEF2C*	
			TFDP1	*IGHA1*	*MALT1*	
			TP53	*IGHV3-23*	*LILRB4*	
			WEE1	*HLA-DRB5*	*CSK*	
				CR1	*TAP1*	

				IGKV3D-11	*HLA-DRB5*	
				TRDC	*CR1*	
				TLR8	*GATA3*	
				EXOSC6	*CD8B*	
				C5	*CD86*	
				C2	*SUSD4*	
				SLA2	*MAP3K7*	
				SUSD4	*SLC11A1*	
				CD46	*NLRP10*	
				CLU	*IFNB1*	
				C8B	*ORAI1*	
				C4B	*ITK*	
				IGHV1OR21-1		
				IGHE		
				C1QB		
				IGHD		
				IGLC1		

Supplementary Table 7. List of genes used to define the six signatures chosen. Defining angiogenesis, hypoxia, DNA repair, cell cycle pathways, B-cell and T-cell immune responses.
