# Supplementary material for: A deep learning model to predict RNA-Seq expression of tumours from whole slide images
Source: Nat Commun. 2020 Aug 3;11:3877. doi: 10.1038/s41467-020-17678-4 (PMC7400514; doi:10.1038/s41467-020-17678-4)
Supplement: Supplementary file 3 — Reporting Summary [file 41467_2020_17678_MOESM3_ESM.pdf]

nature research

Corresponding author(s): Benoit Schmauch, Gilles Wainrib

Last updated by author(s): Jun 24, 2020

Reporting Summary

Nature Research wishes to improve the reproducibility of the work that we publish. This form provides structure for consistency and transparency in reporting. For further information on Nature Research policies, see Authors & Referees and the Editorial Policy Checklist .

Statistics

For all statistical analyses, confirm that the following items are present in the figure legend, table legend, main text, or Methods section.

n/a	Confirmed	
☐	☒	The exact sample size (*n*) for each experimental group/condition, given as a discrete number and unit of measurement
☐	☒	A statement on whether measurements were taken from distinct samples or whether the same sample was measured repeatedly
☐	☒	The statistical test(s) used AND whether they are one- or two-sided<br>*Only common tests should be described solely by name; describe more complex techniques in the Methods section.*
☐	☒	A description of all covariates tested
☐	☒	A description of any assumptions or corrections, such as tests of normality and adjustment for multiple comparisons
☐	☒	A full description of the statistical parameters including central tendency (e.g. means) or other basic estimates (e.g. regression coefficient) AND variation (e.g. standard deviation) or associated estimates of uncertainty (e.g. confidence intervals)
☐	☒	For null hypothesis testing, the test statistic (e.g. *F*, *t*, *r*) with confidence intervals, effect sizes, degrees of freedom and *P* value noted<br>*Give P values as exact values whenever suitable.*
☒	☐	For Bayesian analysis, information on the choice of priors and Markov chain Monte Carlo settings
☐	☒	For hierarchical and complex designs, identification of the appropriate level for tests and full reporting of outcomes
☐	☒	Estimates of effect sizes (e.g. Cohen's *d*, Pearson's *r*), indicating how they were calculated

Our web collection on statistics for biologists contains articles on many of the points above.

Software and code

Policy information about availability of computer code

Data collection

Pathology slides with RNAseq were downloaded from the publicly accessible GDC data portal (https://portal.gdc.cancer.gov/) using the gdc-transfer tool (mentioned in the list below), tile images were downloaded directly from https://zenodo.org/record/1214456#.XpWJbm46--w and prostate adenocarcinoma slides with segmentation mask of the epithelium from http://doi.org/10.5281/zenodo.1485967. No software was used for the latter two datasets.

Data analysis

Softwares Ingenuity Pathway Analysis (IPA 01-14) QuPath==0.1.2 was used, as well as the following python packages (from python 3.7.4):
cffi==1.14.0
colorcorrect==0.9
cryptography==2.8
Cython==0.29.14
decorator==4.3.0
-e git+https://github.com/NCI-GDC/gdc-client.git@1c69ed0c2bfa3c6b3784bca1ab6feaed7a81f6cb#egg=gdc_client
h5py==2.9.0
intervaltree==3.0.2
ipykernel==5.0.0
ipython==7.0.1
ipython-genutils==0.2.0
ipywidgets==7.4.2
jsonschema==2.6.0
jupyter==1.0.0
jupyter-client==5.2.3
jupyter-console==5.2.0
jupyter-contrib-core==0.3.3
jupyter-contrib-nbextensions==0.5.0

```
jupyter-core==4.4.0
jupyter-highlight-selected-word==0.2.0
jupyter-nbextensions-configurator==0.4.0
Keras==2.2.4
Keras-Applications==1.0.6
Keras-Preprocessing==1.0.5
git+https://github.com/src-d/kmcuda.git#subdirectory=src
lxml==4.4.2
matplotlib==3.1.1
mygene==3.0.0
ndg-httpsclient==0.5.0
numba==0.45.1
numpy==1.17.0
openslide-python==1.1.1
pandas==0.23.4
pathlib==1.0.1
Pillow==6.1.0
progressbar2==3.43.1
pyasn1==0.4.3
pyOpenSSL==18.0.0
PyYAML==3.13
requests==2.22.0
scikit-learn==0.21.2
scipy==1.2.1
seaborn==0.9.0
setuptools==45.3.0
skimage==0.16.2
statsmodels==0.9.0
tables==3.5.2
tensorboard==1.14.0
tensorboardX==1.4
tensorflow-estimator==1.14.0
tensorflow-gpu==1.14.0
termcolor==1.1.0
torch==1.4.0
torchvision==0.5.0
tqdm==4.32.2
```

For manuscripts utilizing custom algorithms or software that are central to the research but not yet described in published literature, software must be made available to editors/reviewers. We strongly encourage code deposition in a community repository (e.g. GitHub). See the Nature Research guidelines for submitting code & software for further information.

Data

Policy information about availability of data

All manuscripts must include a data availability statement. This statement should provide the following information, where applicable:

- Accession codes, unique identifiers, or web links for publicly available datasets
- A list of figures that have associated raw data
- A description of any restrictions on data availability

The TCGA dataset is publicly available at the TCGA portal (https://portal.gdc.cancer.gov). Labelled tiles from colorectal cancer samples "100,000 histological images of human colorectal cancer and healthy tissue" and the PESO dataset are publicly available at https://zenodo.org/record/1214456#.XpWJbm46--w and http://doi.org/10.5281/zenodo.1485967 respectively. The Mondor dataset is available from hospital Henri Mondor but restrictions apply to the availability of data, which were used with permission for the current study, and so are not publicly available. The data, or a test subset, may be available from hospital Henri Mondor subject to ethical approvals. Model interpretability can be explored at: https://owkin.com/he2rna-result-visualization/.

Field-specific reporting

Please select the one below that is the best fit for your research. If you are not sure, read the appropriate sections before making your selection.

[x] Life sciences [ ] Behavioural & social sciences [ ] Ecological, evolutionary & environmental sciences

For a reference copy of the document with all sections, see nature.com/documents/nr-reporting-summary-flat.pdf

Life sciences study design

All studies must disclose on these points even when the disclosure is negative.

Sample size	The sample size for transcriptome prediction (n=10,514 slides) was obtained by taking every slide from a primary solid tumor matching with RNAseq data on TCGA. The validation cohorts "100,000 histological images of human colorectal cancer and healthy tissue" (n=100,000 tile images) and PESO (n=62 slides) were used in their integrality. The sample size for MKI67 spatialization (n=369 slides) was obtained by taking every slide with pathologist annotation of tumor area and healthy tissue. We assured that our sample sizes were sufficient to give clinically and statistically robust and significant results by systematically computing p-values to assess the relevance of our results. To strengthen the significance of reported results, we used a restrictive correction method for multiple hypothesis testing (Holm-Sidak, controlling the Family-Wise Error Rate, hence restricting the probability of making any false discovery at all), as opposed to correction schemes more often used, that only control the False Discovery Rate.
Data exclusions	No data were excluded from the study.
Replication	The results have been generated via a python script to guarantee that they can be reproduced easily. Training of the model predicting gene expression was performed in cross-validation, using 5 train-test splits of the data, that showed consistent results (similar correlation per gene and cancer type on average). Results reported in the present work were aggregated over those 5 folds. For each size ratio, MSI prediction was performed on 50 independent splits of the data between the subset used for transcriptome prediction and the subset used for MSI prediction. 10 different cross-validation with 3 folds where furthermore performed on the latter to increase robustness.
Randomization	Randomization of patients for cross-validation was performed completely at random without any stratification.
Blinding	Blinding was not relevant to our study as the training and testing of the model was performed on known and validated gene expression and MSI status (by RNAseq and Immunohistochemistry values respectively). We however ensured that the samples in each datasets were randomly distributed among the training and test sets to ensure to the best of our knowledge an unbiased approach.

Reporting for specific materials, systems and methods

We require information from authors about some types of materials, experimental systems and methods used in many studies. Here, indicate whether each material, system or method listed is relevant to your study. If you are not sure if a list item applies to your research, read the appropriate section before selecting a response.

Materials & experimental systems

n/a	Involved in the study	
☒	☐	Antibodies
☒	☐	Eukaryotic cell lines
☒	☐	Palaeontology
☒	☐	Animals and other organisms
☒	☐	Human research participants
☒	☐	Clinical data

Methods

n/a	Involved in the study	
☒	☐	ChIP-seq
☒	☐	Flow cytometry
☒	☐	MRI-based neuroimaging
